# Strain Distribution at the Composite–Tooth Interface: An In Vitro Study Using Digital Image Correlation and Linear Mixed‐Effects Models

**DOI:** 10.1002/cre2.70495

**Published:** 2026-10-25

**Authors:** Milan Drahos, Jiri Benes, Gerald Stach, Jan Zeman, Jan Bernard Matschke, Christiane Keil, Ludek Heller, Michaela Buckova

**Affiliations:** ^1^ Department of Medical Statistics, First Faculty of Medicine, Institute of Biophysics and Informatics Charles University in Prague Prague Czech Republic; ^2^ Faculty of Health Studies of the Technical University of Liberec Liberec Czech Republic; ^3^ Department of Oral and Maxillofacial Surgery Faculty of Medicine and University Hospital Carl Gustav Carus, TUD Dresden University of Technology Dresden Germany; ^4^ Department of Orthodontics Faculty of Medicine and University Hospital Carl Gustav Carus, TUD Dresden University of Technology Dresden Germany; ^5^ Institute of Physics of the Czech Academy of Sciences Prague Czech Republic

**Keywords:** bulk‐fill composites, digital image correlation, interfacial strain, linear mixed‐effects models, polymerization shrinkage

## Abstract

**Objectives:**

To compare polymerization‐induced interfacial strain among two high‐viscosity bulk‐fill resin composites and a conventional reference composite using digital image correlation (DIC), and to evaluate regional differences in strain.

**Materials and Methods:**

Thirty extracted human molars were prepared and randomly allocated to three restorative materials (*n* = 10 per group): Filtek One Bulk Fill, Tetric PowerFill, and Tetric EvoCeram as the conventional reference composite. Polymerization‐induced interfacial strain was assessed using DIC. Eight strain values were exported for each of three predefined cavity regions (mesial, central, and distal) per tooth, yielding 720 values from 30 independent specimens. Data were analyzed using linear mixed‐effects models with material and cavity region as fixed effects and random intercepts for tooth and tooth‐by‐region. Pairwise comparisons were performed using estimated marginal means with Tukey adjustment.

**Results:**

Mean strain was lowest for Filtek One Bulk Fill (0.68% ± 0.13%), followed by Tetric PowerFill (0.74% ± 0.17%) and Tetric EvoCeram (1.12% ± 0.23%). The final additive model yielded significant material (*F*[2, 715] = 419.34, *p* < 0.001) and region effects (*F*[2, 715] = 8.97, *p* < 0.001). Tukey‐adjusted comparisons indicated differences between all materials, including Filtek One Bulk Fill versus Tetric PowerFill (adjusted *p* = 0.0054). The interaction did not improve model fit. Regional means were identical across teeth within each material group, and both random‐intercept variances were zero, resulting in a singular fit and limiting inferential precision.

**Conclusions:**

Under the present in vitro conditions, the analyzed dataset showed lower interfacial strain for both tested bulk‐fill composites than for Tetric EvoCeram, with a small difference between the bulk‐fill materials. Interpretation is constrained by the repeated regional means and singular model fit, and clinical relevance remains undetermined. The implemented DIC workflow provides a basis for further methodological refinement.

**Abbreviations**
AIC: Akaike information criterion
DIC: digital image correlation
LMM: linear mixed model
SD: standard deviation

## 1 Introduction

Polymerization shrinkage is an inherent characteristic of resin‐based dental composites and remains an important factor influencing the performance and longevity of adhesive restorations. During light‐activated polymerization, monomer conversion is accompanied by volumetric contraction. When shrinkage is constrained by adhesion to the cavity walls, stresses may develop at the composite–tooth interface, potentially affecting marginal integrity and interfacial stability. The magnitude and distribution of these stresses depend not only on the material itself but also on cavity geometry, bonding conditions, and polymerization kinetics. Recent investigations of contemporary resin composites continue to demonstrate substantial material‐dependent differences in polymerization shrinkage and shrinkage‐stress behavior (Szczesio‐Wlodarczyk et al. 2024a, 2024b).

The adhesive interface plays an important role in the mechanical coupling between the restorative composite and dental tissues and may therefore influence the transfer of polymerization‐induced deformation to the surrounding tooth structure. Adhesive performance depends on the bonding strategy, substrate conditioning, and characteristics of the adhesive layer, making standardization of the bonding protocol particularly important when interfacial strain is investigated (Perdigão et al. 2021).

Bulk‐fill resin composites were introduced to simplify restorative procedures by allowing placement in thicker increments while maintaining adequate polymerization throughout the restoration. Contemporary bulk‐fill materials incorporate modifications of the resin matrix, filler system, translucency, and photoinitiator technology designed to improve depth of cure and modulate polymerization kinetics. Recent evidence indicates that adequate degree of conversion can be achieved with contemporary bulk‐fill materials, although performance remains dependent on material composition, increment thickness, and curing conditions (Hatipoğlu et al. 2024).

Importantly, the simplified placement protocol does not appear to compromise clinical performance. Recent systematic reviews and long‐term randomized clinical studies have reported clinical outcomes of posterior bulk‐fill restorations comparable to those obtained with conventional incremental techniques (de Menezes et al. 2025; Kunz et al. 2022; Loguercio et al. 2023). In a 5‐year randomized controlled trial, Loguercio et al. found similar overall clinical behavior for posterior restorations placed using bulk‐fill and incremental techniques. Experimental studies evaluating marginal adaptation likewise indicate that high‐viscosity bulk‐fill composites can achieve marginal integrity comparable to conventional composites after thermomechanical loading (Hamza et al. 2022).

Despite these favorable clinical and laboratory findings, the mechanisms by which polymerization‐induced deformation is transferred to the composite–tooth interface remain incompletely characterized. Conventional methods used to investigate polymerization shrinkage or shrinkage stress generally provide global or point‐based measurements and therefore offer limited information about spatially heterogeneous deformation. Digital image correlation (DIC), in contrast, is a non‐contact optical technique that enables full‐field measurement and visualization of displacement and strain. Although well established in experimental mechanics, its application in dental materials research remains comparatively limited, as summarized by Yoon et al. (2021).

A particularly relevant application of DIC to dental composite polymerization was reported by Martinsen et al. (2013), who used three‐dimensional DIC to characterize full‐field polymerization shrinkage in bar‐shaped composite specimens. Their study demonstrated that polymerization‐induced deformation is spatially heterogeneous, with peak shrinkage occurring below the light‐exposed surface rather than being uniformly distributed throughout the material. This work established the ability of DIC to resolve complex three‐dimensional shrinkage patterns that cannot be adequately characterized by conventional global measurements. However, the analysis focused on deformation within the composite material itself in an unconstrained specimen geometry, rather than on deformation transmitted across a bonded composite–tooth interface.

The clinically relevant adhesive interface therefore represents a distinct experimental problem. In a restored tooth, polymerization shrinkage is constrained by adhesion to surrounding dental tissues, and the resulting interfacial deformation may vary spatially according to local cavity geometry and boundary conditions. Despite increasing interest in DIC for dental materials research (Chojnacka‐Brożek et al. 2024; Yoon et al. 2021), limited evidence is available on polymerization‐induced strain measured directly along the composite–tooth interface, particularly when different classes of restorative composites are compared within a standardized cavity configuration. Moreover, regional measurements obtained from the same tooth are inherently correlated and require statistical methods that account for this hierarchical data structure.

The present study therefore combined DIC with linear mixed‐effects modeling to evaluate polymerization‐induced strain at the composite–tooth interface in standardized Class I restorations fabricated using two high‐viscosity bulk‐fill composites and one conventional reference composite. The objectives were to (a) characterize the spatial distribution of interfacial strain during polymerization and (b) quantify the effects of composite material and cavity region while accounting for repeated regional measurements within individual specimens. By shifting the DIC analysis from shrinkage within an isolated composite specimen to strain developing at the bonded composite–tooth interface, the study extends previous full‐field approaches toward a clinically more representative restorative configuration.

## 2 Materials and Methods

### 2.1 Specimen Preparation

Thirty extracted human molars were obtained following routine dental extractions. All specimens were anonymized; therefore, patient demographic information, including age and sex, was unavailable. Before cavity preparation, caries status and structural integrity were assessed by visual inspection under optical magnification (approximately 22.5×). Carious lesions were classified as Ekstrand scores 2–3. All specimens also underwent cone‐beam computed tomography (CBCT) to exclude teeth with existing restorations, extensive structural defects, or detectable cracks.

After cleaning, the teeth were stored in 0.1% thymol solution at approximately 4°C for 1 year before use. Standardized Class I cavities with target dimensions of 4 × 4 × 3 mm were prepared using a diamond bur under continuous water cooling. For DIC measurements, the specimens were embedded in epoxy resin (MC 35.1/W21) and mounted in custom stainless‐steel holders. Four M3 screws arranged in two orthogonal pairs were used to stabilize the specimens and minimize movement during image acquisition.

The specimens were allocated to three restorative material groups, with 10 teeth per group.

### 2.2 Adhesive and Restorative Procedure

After cavity preparation, selective caries removal was guided by Caries Detector (Cerkamed, Stalowa Wola, Poland). Peripheral carious dentin was completely removed to hard dentin using green‐ring carbide burs in a low‐speed handpiece, whereas selective caries removal at the pulpal wall was performed according to the manufacturer's color indicator to preserve affected dentin where appropriate. The cavities were subsequently rinsed with chlorhexidine solution and gently air‐dried.

Adhesive bonding was performed using OptiBond Solo Plus (Kerr Corporation, Orange, CA, USA), a two‐step etch‐and‐rinse adhesive system. Enamel and dentin surfaces were etched with 37.5% phosphoric acid (Kerr Gel Etchant) for 15 s and thoroughly rinsed with water for approximately 15–20 s. The surfaces were gently air‐dried for approximately 5 s, avoiding dentin desiccation. The adhesive was applied using a disposable applicator with a light brushing motion for 15 s, gently air‐thinned for 3 s to obtain a uniform layer and prevent pooling, and light‐cured for 20 s using a 3 M Elipar DeepCure‐L LED curing light (3M ESPE, Seefeld, Germany).

Following adhesive curing, a thin layer of flowable resin composite Tetric EvoFlow (Ivoclar Vivadent, Schaan, Liechtenstein) was applied to the cavity floor. The restorations were then completed using Filtek One Bulk Fill Restorative (3M ESPE, Seefeld, Germany), Tetric PowerFill, or Tetric EvoCeram (both Ivoclar Vivadent, Schaan, Liechtenstein), according to the assigned experimental group.

### 2.3 Light‐Curing Procedure

All adhesive and restorative materials were light‐cured using a 3M Elipar DeepCure‐L LED curing light (3M ESPE, Seefeld, Germany), with a manufacturer‐specified nominal irradiance of approximately 1470 mW/cm^2^ and a wavelength range of 430–480 nm. Irradiance was not independently verified using a radiometer during the experiment; therefore, the reported irradiance represents the nominal output rather than an experimentally measured value. The light guide was positioned perpendicular to the restoration surface and maintained in direct contact with, or as close as possible to, the material during light curing.

The OptiBond Solo Plus adhesive layer was light‐cured for 20 s. The flowable resin composite Tetric EvoFlow was applied to the cavity floor at a thickness of approximately 0.5 mm and light‐cured for 20 s. Filtek One Bulk Fill and Tetric PowerFill were placed using a bulk‐fill technique within the prepared cavities and light‐cured for 20 and 10 s, respectively. Tetric EvoCeram was placed in approximately 1.5‐mm increments, with each increment light‐cured for 10 s. The same curing light and light‐guide positioning were used for all groups.

### 2.4 DIC

Polymerization‐induced interfacial strain was assessed using a non‐contact DIC system (Mercury RT, Sobriety, Czech Republic). Before image acquisition, a stochastic high‐contrast speckle pattern was applied to the specimen surface to enable optical displacement tracking. Pattern quality was visually inspected for contrast, randomness, and coverage of the evaluated area. Images acquired before light curing of the restorative composite served as the reference configuration for subsequent displacement and strain calculations. Image acquisition was performed during polymerization without external mechanical loading.

For each tooth, three predefined cavity regions were evaluated: mesial, central, and distal. Images acquired before light curing of the restorative composite served as the reference configuration for subsequent displacement and strain calculations. Image acquisition was performed during polymerization without external mechanical loading.

For each tooth, three predefined cavity regions were evaluated: mesial, central, and distal. The labels G1–G8 were assigned separately within each region, yielding eight exported strain values per region, 24 values per tooth, and 720 values from 30 independent teeth. The evaluated quantity was the first principal strain. Measurement locations are illustrated in Figure 1.

**Figure 1:**
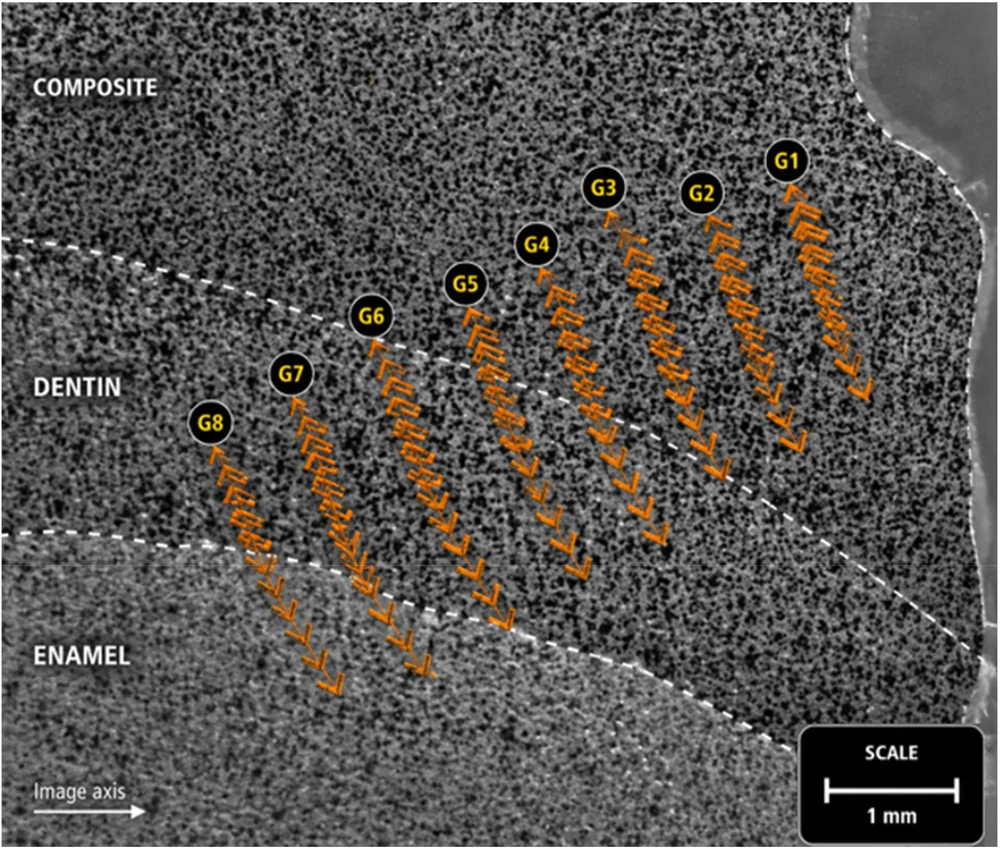
Representative DIC micrograph of the composite–tooth interface. Measurement locations G1–G8 indicate the predefined positions used for displacement analysis, with arrows representing the corresponding displacement vectors. The approximate boundaries between composite, dentin, and enamel are indicated by dashed lines. The image axis and a 1‐mm scale bar are shown for orientation.

Images had a resolution of approximately 2400 × 2000 pixels, corresponding to a ground sampling distance of approximately 5–15 µm per pixel. The system was calibrated using a planar calibration target. Strain fields were computed using subset‐based correlation with a subset size of 15–25 pixels and a step size of 5–10 pixels, resulting in an effective spatial resolution of approximately 0.1–0.3 mm and a strain sensitivity on the order of 10^−4^. Strain was expressed as the first principal Green–Lagrange strain, and each measurement position represented a locally averaged strain value within a defined evaluation area rather than a single‐pixel measurement. DIC measurement uncertainty and repeatability were not independently quantified.

### 2.5 Statistical Analysis

All statistical analyses were performed using R version 4.3. The dataset comprised 720 exported, locally averaged strain values from 30 independent teeth, with eight values within each of three predefined cavity regions (mesial, central, and distal) per tooth. These within‐tooth observations did not increase the number of independent experimental specimens. Descriptive statistics are presented as mean ± standard deviation.

Linear mixed‐effects models were fitted using the lme4 package. Interfacial strain was the dependent variable, and material and cavity region were included as fixed effects. The initial model additionally included the Material × Region interaction. Random intercepts for tooth and tooth‐by‐region were specified to represent the hierarchical structure of the dataset. Models with and without the interaction were compared using the Akaike Information Criterion (AIC) after fitting by maximum likelihood. Because the additive model had the lower AIC, it was selected for the reported analysis:

Strain~Material+Region+(1|Tooth)+(1|Tooth:Region).

Model comparisons are presented in Table S1.

Fixed effects were tested using the *lmer* Test package with the Satterthwaite approximation for denominator degrees of freedom. Pairwise comparisons were calculated using estimated marginal means from the *emmeans* package, with Tukey adjustment for multiple comparisons. Effect sizes are reported as partial *η* ^2^. Fixed‐effect estimates, standard errors, and 95% confidence intervals (CIs) are provided in Table S2.

Model diagnostics included Q–Q plots, residuals‐versus‐fitted plots, and the Shapiro–Wilk test of residuals. Random‐effect variance estimates and model singularity were also examined. Both random‐intercept variances were estimated at zero, resulting in a singular fit; therefore, specification of the random effects alone did not establish adequate adjustment for within‐tooth dependence.

A leave‐one‐tooth‐out analysis was performed to assess the influence of individual specimens on the estimated material effects. This analysis did not independently validate the DIC processing workflow or resolve the repeated regional means across specimens. The significance threshold was set at *p* < 0.05. No a priori sample‐size calculation was performed.

## 3 Results

Descriptive statistics for interfacial strain by composite material are summarized in Table 1. In the exported dataset, both tested bulk‐fill composites had lower mean strain values than the conventional reference composite, Tetric EvoCeram, in each evaluated cavity region.

**Table 1:** Interfacial strain according to the composite material.

Composite material	Interfacial strain, % (mean ± SD)
Filtek one bulk fill	0.68 ± 0.13^a^
Tetric powerFill	0.74 ± 0.17^b^
Tetric EvoCeram	1.12 ± 0.23ᶜ

*Note:* Values are presented as mean ± SD. The superscript letters (a–c) indicate statistically significant pairwise differences between the composite materials. Materials with different superscript letters differ significantly based on Tukey‐adjusted post‐hoc comparisons from the final additive model.

### 3.1 Interfacial Strain by Material

The estimated marginal mean (EMM) strain was lowest for Filtek One Bulk Fill (0.683%; model‐based 95% CI 0.659–0.707), followed by Tetric PowerFill (0.740%; 95% CI 0.716–0.764) and Tetric EvoCeram (1.123%; 95% CI 1.099–1.147). The final additive model yielded a statistically significant material effect, *F*(2, 715) = 419.34, *p* < 0.001, partial *η* ^2^ = 0.540. The fixed effects of the final additive linear mixed‐effects model are summarized in Table 2.

**Table 2:** Fixed effects of the final additive linear mixed‐effects model for interfacial strain.

Effect	Numerator df	Denominator df	*F*	*p* value	Partial *η* ^2^
Material	2	715	419.34	< 0.001	0.540
Region	2	715	8.97	< 0.001	0.024

*Note:* The final model included composite material and measurement region as fixed effects and tooth and tooth‐by‐region as random effects. The material × region interaction was excluded from the final model because its inclusion did not improve model fit.

Abbreviations: df, degrees of freedom; *F*, *F*‐statistic; *η* ^2^ *p*, partial eta squared.

Tukey‐adjusted comparisons from this model indicated lower strain for Filtek One Bulk Fill than for Tetric PowerFill (mean difference −0.0567 percentage points; 95% CI −0.0977 to −0.0157; adjusted *p* = 0.0054). Both bulk‐fill materials also had lower estimated strain than Tetric EvoCeram: the mean difference was −0.4400 percentage points for Filtek One Bulk Fill (95% CI −0.4810 to −0.3990) and −0.3833 percentage points for Tetric PowerFill (95% CI −0.4243 to −0.3423), with both adjusted *p* < 0.0001. These inferential outputs are subject to the dataset and model limitations described below.

### 3.2 Regional Strain Distribution

The final additive model yielded a statistically significant region effect, *F*(2, 715) = 8.97, *p* = 0.000142, partial *η* ^2^ = 0.024. The EMM strain was 0.813% in the mesial region (model‐based 95% CI 0.790–0.837), 0.850% in the central region (95% CI 0.827–0.873), and 0.883% in the distal region (95% CI 0.860–0.907).

Tukey‐adjusted comparisons indicated higher strain in the distal than in the mesial region (mean difference 0.0700 percentage points; adjusted *p* = 0.0002). The mesial–central and central–distal comparisons did not reach statistical significance after adjustment (adjusted *p* = 0.0765 and 0.1173, respectively).

Adding the material × region interaction did not improve model fit (likelihood‐ratio test: *χ* ^2^[4] = 0.929, *p* = 0.920), and AIC favored the additive model. The interaction was therefore excluded from the final analysis.

### 3.3 Model Performance and Variability

The final additive model converged but produced a singular fit. The estimated random‐intercept variances were zero for both tooth and tooth × region. The residual variance was 0.03279 (SD = 0.1811). Variance components are presented in Table S3. The marginal *R* ^2^ was 0.544; a conditional *R* ^2^ was not reported.

Examination of the exported dataset showed that the mean of the eight values within each tooth–region combination was identical across teeth within the same material group, to numerical precision, despite variation among individual values. The zero estimated random‐effect variances therefore cannot be interpreted as evidence that biological variability between teeth was absent. This unresolved data‐processing issue limits the interpretation of the model‐based standard errors, CIs, and *p*‐values.

The leave‐one‐tooth‐out analyses also produced singular fits. These analyses assessed the influence of individual specimens but did not resolve the repeated regional means or establish the adequacy of adjustment for within‐tooth dependence. The dataset contained 720 observations from 30 independent experimental specimens.

The Shapiro–Wilk test did not indicate a statistically significant departure from residual normality (*W* = 0.9962, *p* = 0.0812). Visual inspection of the Q–Q plot showed mild deviations in the tails. The residuals‐versus‐fitted plot showed no pronounced systematic trend, although residual dispersion was greater at higher fitted values (Figure 2). These diagnostics do not resolve the uncertainty concerning the processing and dependence structure of the observations.

**Figure 2:**
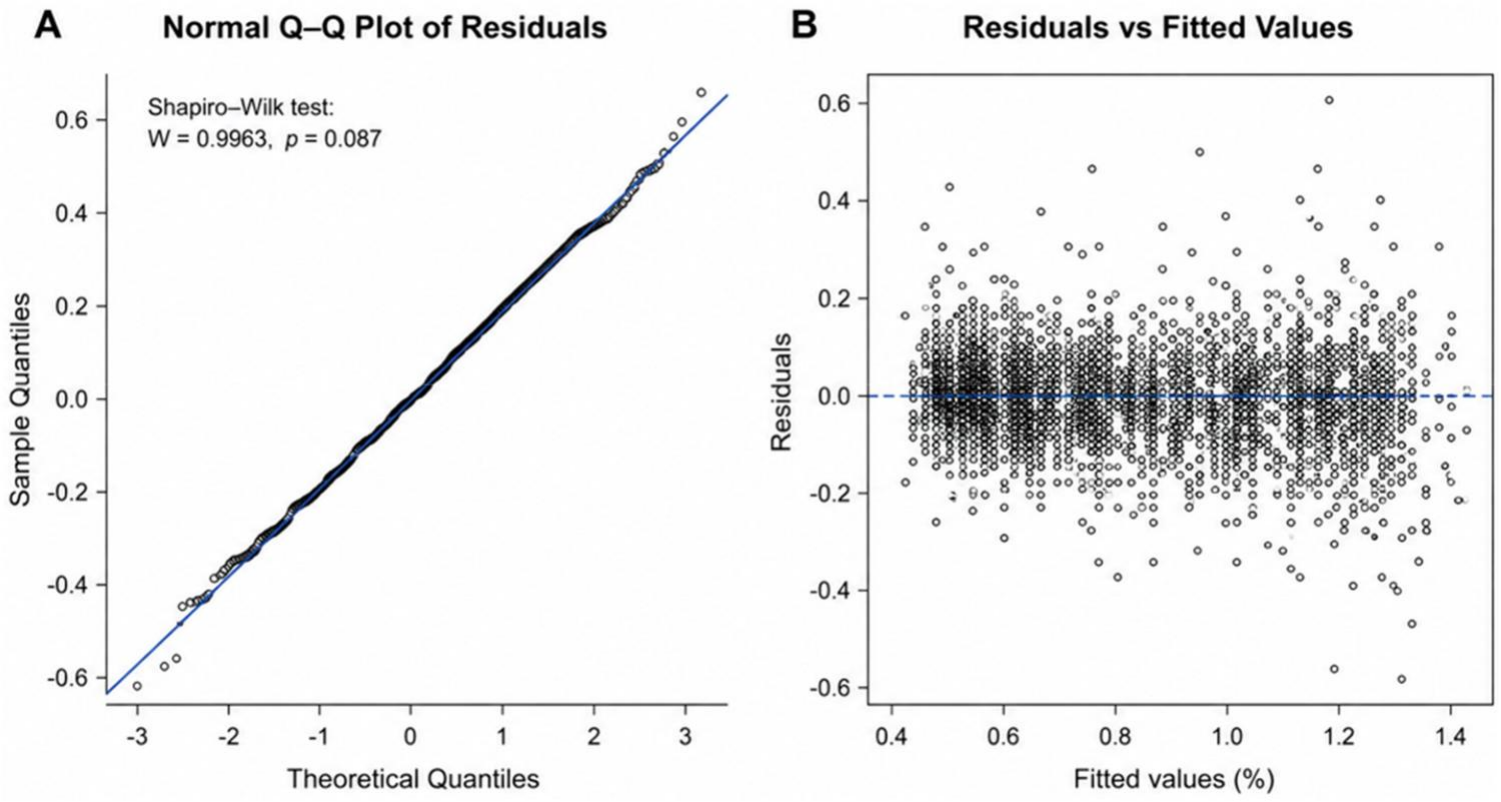
Model diagnostics of the linear mixed‐effects analysis. (A) Normal Q–Q plot of model residuals. (B) Residuals plotted against fitted values. The Q–Q plot showed only mild deviations in the distribution tails, while the residuals‐versus‐fitted plot revealed no pronounced systematic trend, although residual dispersion was somewhat greater at higher fitted values. The Shapiro–Wilk test did not indicate a significant deviation from residual normality (*W* = 0.9962, *p* = 0.0812).

## 4 Discussion

This study investigated polymerization‐induced strain at the composite–tooth interface using DIC combined with linear mixed‐effects modeling. The tested high‐viscosity bulk‐fill composites exhibited significantly lower interfacial strain than the conventional reference composite. A significant regional effect was also observed, with higher strain in the distal region. No statistically significant material × region interaction was detected, providing no evidence of material‐dependent regional variation within the current sample.

### 4.1 Material‐Dependent Polymerization Behavior

The reduced interfacial strain observed for the two tested bulk‐fill composites may reflect different formulation‐specific strategies for modulating polymerization kinetics and stress development. Filtek One Bulk Fill contains high‐molecular‐weight AUDMA and a proprietary addition–fragmentation monomer (AFM), which are intended to reduce polymerization shrinkage and facilitate stress relaxation during network formation (Par et al. 2022; Szebeni et al. 2024). Tetric PowerFill employs a different resin formulation containing β‐allyl sulfone as an addition–fragmentation chain‐transfer (AFCT) agent, together with a photoinitiator system comprising camphorquinone/amine and Ivocerin. These components can modify polymerization kinetics and network formation and may thereby influence the development of polymerization‐induced stress (Marovic et al. 2025; Par et al. 2022; Szebeni et al. 2024).

In the analyzed dataset, both bulk‐fill composites exhibited lower interfacial strain than the conventional reference composite. The final additive model yielded a lower estimated strain for Filtek One Bulk Fill than for Tetric PowerFill (mean difference 0.057 percentage points, 95% CI 0.016–0.098, adjusted *p* = 0.0054). This difference was small compared with the corresponding differences from Tetric EvoCeram (0.440 and 0.383 percentage points, respectively). Its interpretation should take account of the processing and statistical limitations described below, as well as the absence of independently quantified measurement uncertainty or an established threshold for clinical relevance.

The absolute strain values provide a basis for comparison with previous DIC‐based investigations of deformation in resin composites (Chojnacka‐Brożek et al. 2024; Martinsen et al. 2013; Yoon et al. 2021). Direct quantitative comparison remains difficult because measured strain depends on material composition, specimen geometry, bonding constraints, measurement location, dimensionality of the DIC approach, and image‐processing parameters. Martinsen et al. demonstrated heterogeneous three‐dimensional shrinkage fields within composite specimens, whereas the present study focused on interfacial strain in bonded Class I restorations. These studies therefore provide methodological context rather than directly equivalent measurements.

### 4.2 Role of the Adhesive Interface

The adhesive interface represents an additional factor that may influence the transfer of polymerization‐induced deformation from the restorative material to the surrounding tooth structure. Bonding strategy, substrate conditioning, and the properties of the adhesive layer can affect the mechanical coupling between the composite and enamel or dentin and may therefore modify the resulting interfacial deformation (Perdigão et al. 2021). In the present study, the same adhesive protocol was applied to all specimens, thereby maintaining this factor constant across the three material groups. Nevertheless, the observed strain patterns should be interpreted within the context of the specific adhesive protocol used, and their transferability to other adhesive systems or bonding strategies remains to be established.

### 4.3 Regional Strain Distribution

A significant effect of cavity region on interfacial strain was observed, with the distal region showing higher strain values across the tested materials. This finding indicates that local cavity geometry and boundary conditions may influence polymerization‐induced deformation. Previous investigations have demonstrated that variations in configuration factor (C‐factor) and local constraint can affect stress development during composite polymerization (Han et al. 2016).

Several mechanisms may contribute to the elevated strain observed in the distal region. In addition to differences in local cavity geometry and constraint, spatial variations in light delivery may influence polymerization kinetics across the restoration. Even under standardized positioning of the curing light, small differences in local tip‐to‐surface distance, angulation, or accessibility may result in non‐uniform irradiance within the cavity (Marovic et al. 2025; Par et al. 2022). Furthermore, polymerization shrinkage is influenced by the configuration of bonded and unbonded surfaces and may therefore produce asymmetric deformation and localized strain concentrations at the composite–tooth interface. The higher distal strain should therefore not be attributed exclusively to C‐factor effects but may reflect the combined influence of cavity geometry, light‐delivery conditions, polymerization dynamics, and experimental boundary conditions. The present experimental design does not allow the relative contribution of these mechanisms to be distinguished. Future studies combining full‐field DIC measurements with finite element modeling may help clarify the mechanical basis of regional strain development and distinguish material‐related effects from those arising from cavity geometry and experimental boundary conditions (Drahoš et al. 2026).

### 4.4 Methodological Strengths

A methodological strength of this study is the use of DIC to assess spatially distributed deformation at the composite–tooth interface. This technically demanding approach required coordinated specimen preparation, stable fixation, surface‐pattern application, and optical image acquisition during polymerization. Evaluation of predefined measurement locations across three cavity regions enabled comparison of local strain values among the tested restorative materials under a standardized experimental protocol.

The statistical analysis specified random intercepts for tooth and tooth‐by‐region to reflect the hierarchical study design. Both random‐effect variances were estimated at zero, resulting in a singular fit. The implications of this finding and the repeated regional means across specimens are addressed in the Limitations section.

### 4.5 Clinical Implications

From a clinical perspective, lower polymerization‐induced interfacial strain may contribute to improved marginal integrity by reducing deformation and stress transfer across the adhesive interface. However, the present study assessed polymerization‐induced strain under controlled in vitro conditions and did not directly evaluate marginal gap formation, microleakage, bond strength, or long‐term restoration survival. Therefore, the observed differences in interfacial strain should not be interpreted as direct evidence of superior clinical performance.

Previous laboratory and clinical studies have reported satisfactory marginal adaptation and clinical performance of bulk‐fill restorations, but the relationship between individual laboratory material properties and clinical outcomes is complex. Systematic evaluations have shown that correlations between laboratory mechanical or physicochemical tests and clinical performance are generally limited, emphasizing that in vitro strain measurements represent only one component of restorative behavior (Heintze et al. 2015, 2017).

The present findings contribute to the experimental characterization of interfacial deformation among the tested materials. Further studies directly assessing marginal integrity, bond durability, and long‐term restoration performance are needed to determine their clinical significance.

### 4.6 Limitations and Future Perspectives

This in vitro study did not include thermomechanical aging or functional loading and therefore does not address long‐term clinical performance. Only one conventional reference composite and one adhesive protocol were investigated, limiting generalizability beyond the tested materials and bonding conditions.

The dataset comprised 720 exported strain values from 30 independent teeth (*n* = 10 per material group). Although individual values varied, regional means were identical across teeth within each material group. The exact cause could not be established from the available processing and export information. Both random‐intercept variances were estimated at zero, resulting in a singular fit. This pattern limits the interpretation of between‐tooth variability and the precision of the model‐based comparisons; the leave‐one‐tooth‐out analysis did not resolve it.

DIC measurement uncertainty and repeatability were not independently quantified. Consequently, the small difference between Filtek One Bulk Fill and Tetric PowerFill (0.057 percentage points) cannot be assessed against measurement uncertainty, and its clinical relevance remains undetermined. No a priori sample‐size calculation was performed. Further investigations should include dedicated uncertainty and repeatability assessments and retain the original processing settings and intermediate outputs to enable verification of the exported values.

## 5 Conclusion

In the analyzed dataset, both tested high‐viscosity bulk‐fill composites showed lower polymerization‐induced interfacial strain than the conventional reference composite, Tetric EvoCeram. The final additive model also indicated a small difference between Filtek One Bulk Fill and Tetric PowerFill and a smaller regional effect. Inclusion of a material × region interaction did not improve model fit. These findings should be interpreted within the limitations of the exported dataset and singular model fit; their clinical relevance remains to be established.

The study implemented a workflow combining standardized specimen preparation, DIC acquisition during polymerization, regional strain evaluation, and statistical analysis. Its application highlighted challenges in data‐processing documentation and specimen‐level interpretation, providing a basis for methodological refinement and future studies incorporating explicit processing checks and repeatability assessment.

## Author Contributions

**Milan Drahos:** investigation, methodology, data curation, writing – original draft. **Jiri Benes:** methodology, formal analysis, software, writing – review and editing. **Gerald Stach:** investigation, methodology, writing – review and editing. **Jan Zeman:** formal analysis, methodology, writing – review and editing. **Jan Bernard Matschke:** writing – review and editing including interpretation of the results and critical revision of the manuscript. **Christiane Keil:** methodology, validation, writing – review and editing. **Ludek Heller:** methodology, formal analysis, writing – review and editing. **Michaela Buckova:** conceptualization, methodology, supervision, project administration, writing – original draft, writing – review and editing. All authors reviewed and approved the final version of the manuscript.

## Funding

The authors have nothing to report.

## Ethics Statement

The teeth were obtained following routine dental extractions and selected from an existing collection maintained for dental student teaching. The selected specimens had not previously been used for teaching procedures and had not undergone student preparation or restoration. Written patient consent covered both the extraction and the subsequent use of the extracted teeth for scientific purposes. All specimens were anonymized before inclusion in the study, and no patient‐identifying information was available to the researchers. The consent documentation can be made available to the journal upon request.

## Conflicts of Interest

The authors declare no conflicts of interest.

## Supporting information


Supporting File 1

Supporting File 2

Supporting File 3

## Data Availability

The data supporting the findings of this study are provided in the Supporting Information. Additional information is available from the corresponding author upon reasonable request.
