# Supplementary material for: Strain Distribution at the Composite–Tooth Interface: An In Vitro Study Using Digital Image Correlation and Linear Mixed‐Effects Models
Source: Clin Exp Dent Res. 2026 Oct 25;12(5):e70495. doi: 10.1002/cre2.70495 (PMC13696018; doi:10.1002/cre2.70495)
Supplement: Supplementary file 1 [file CRE2-12-e70495-s002.docx]

**Supplementary**

**Supplementary Table S1. Comparison of alternative model structures**

| **Model structure** | **df** | **AIC** | **ΔAIC** |
| --- | --- | --- | --- |
| Material + Region | 8 | −406.429 | 0.000 |
| Material × Region | 12 | −399.358 | 7.071 |
| Material | 6 | −392.581 | 13.848 |
| Region | 6 | −280.936 | 125.494 |
| Null model | 4 | −267.831 | 138.598 |

**Supplementary Table S2. Fixed-effect coefficient estimates from the final additive linear mixed-effects model with 95% confidence intervals**

| **Effect** | **Estimate (β)** | **SE** | **df** | **95% CI** | **p-value** |
| --- | --- | --- | --- | --- | --- |
| Intercept | 0.648 | 0.015 | 715 | 0.618 to 0.678 | < 0.001 |
| Tetric PowerFill vs Filtek | 0.057 | 0.017 | 715 | 0.024 to 0.089 | < 0.001 |
| Tetric EvoCeram vs Filtek | 0.440 | 0.017 | 715 | 0.408 to 0.472 | < 0.001 |
| Central vs Mesial | 0.037 | 0.017 | 715 | 0.004 to 0.069 | 0.027 |
| Distal vs Mesial | 0.070 | 0.017 | 715 | 0.038 to 0.102 | < 0.001 |

β, fixed-effect coefficient estimate; SE, standard error; CI, confidence interval. The intercept represents the estimated mean interfacial strain for Filtek One Bulk Fill in the Mesial region. Coefficients for composite material and region represent differences relative to the corresponding reference categories.

**Supplementary Table S3. Variance components of the hierarchical model**

| **Component** | **Variance** | **SD** |
| --- | --- | --- |
| Tooth | 0.000000 | 0.000000 |
| Tooth × Region | 0.000000 | 0.000000 |
| Residual | 0.03279 | 0.1811 |

*The random-intercept variances for Tooth and Tooth × Region were estimated at zero, resulting in a singular fit. Variance components are reported for the final additive model.*
